# Selection for Phage Resistance Reduces Virulence of Shigella flexneri

**DOI:** 10.1128/AEM.01514-21

**Published:** 2022-01-25

**Authors:** Kaitlyn E. Kortright, Rachel E. Done, Benjamin K. Chan, Valeria Souza, Paul E. Turner

**Affiliations:** a Program in Microbiology, Yale School of Medicine, New Haven, Connecticut, USA; b Department of Ecology and Evolutionary Biology, Yale University, New Haven, Connecticut, USA; c Departamento de Ecologia Evolutiva, Instituto de Ecologia, Universidad Nacional Autonoma de Mexico, Mexico City, Mexico; Unversidad de los Andes

**Keywords:** phage, virulence, resistance, evolution, trade-off

## Abstract

There is an increasing interest in phage therapy as an alternative to antibiotics for treating bacterial infections, especially using phages that select for evolutionary trade-offs between increased phage resistance and decreased fitness traits, such as virulence, in target bacteria. A vast repertoire of virulence factors allows the opportunistic bacterial pathogen Shigella flexneri to invade human gut epithelial cells, replicate intracellularly, and evade host immunity through intercellular spread. It has been previously shown that OmpA is necessary for the intercellular spread of S. flexneri. We hypothesized that a phage which uses OmpA as a receptor to infect S. flexneri should select for phage-resistant mutants with attenuated intercellular spread. Here, we show that phage A1-1 requires OmpA as a receptor and selects for reduced virulence in S. flexneri. We characterized five phage-resistant mutants by measuring phenotypic changes in various traits: cell-membrane permeability, total lipopolysaccharide (LPS), sensitivity to antibiotics, and susceptibility to other phages. The results separated the mutants into two groups: R1 and R2 phenotypically resembled *ompA* knockouts, whereas R3, R4, and R5 were similar to LPS-deficient strains. Whole-genome sequencing confirmed that R1 and R2 had mutations in *ompA*, while R3, R4, and R5 had mutations in the LPS inner-core biosynthesis genes *gmhA* and *gmhC*. Bacterial plaque assays confirmed that all the phage-resistant mutants were incapable of intercellular spread. We concluded that selection for S. flexneri resistance to phage A1-1 generally reduced virulence (i.e., intercellular spread), but this trade-off could be mediated by mutations either in *ompA* or in LPS-core genes that likely altered OmpA conformation.

**IMPORTANCE** Shigella flexneri is a facultative intracellular pathogen of humans and a leading cause of bacillary dysentery. With few effective treatments and rising antibiotic resistance in these bacteria, there is increasing interest in alternatives to classical infection management of S. flexneri infections. Phage therapy poses an attractive alternative, particularly if a therapeutic phage can be found that results in an evolutionary trade-off between phage resistance and bacterial virulence. Here, we isolate a novel lytic phage from water collected in Cuatro Cienegas, Mexico, which uses the OmpA porin of S. flexneri as a receptor. We use phenotypic assays and genome sequencing to show that phage A1-1 selects for phage-resistant mutants which can be grouped into two categories: OmpA-deficient mutants and LPS-deficient mutants. Despite these underlying mechanistic differences, we confirmed that naturally occurring phage A1-1 selected for evolved phage resistance which coincided with impaired intercellular spread of S. flexneri in a eukaryotic infection model.

## INTRODUCTION

As a facultative intracellular pathogen and a causative agent of bacillary dysentery, Shigella flexneri is a Gram-negative bacterium of medical importance. Fecal-oral propagation is the predominant mode of transmission for these bacteria, and usually occurs via contaminated drinking water (1). Bacteria rapidly traverse the gastrointestinal tract to invade colonic epithelial cells. Upon invasion, both chromosomally-encoded and plasmid-encoded virulence factors enable S. flexneri to overcome host immune responses, replicate intracellularly, and spread to neighboring epithelial cells (2). In particular, the intercellular spread of S. flexneri is a virulence trait that damages colonic epithelial cells, destroying barrier function and resulting in severe shigellosis and dysentery.

Shigellosis is a major public health problem in low-income countries that lack dependable water sanitation, and this disease contributes to approximately 1.3 million deaths annually (3). Children under the age of five account for almost 70% of the total mortality, primarily for cases in sub-Saharan Africa and southern Asia (4). Antibiotic treatment for shigellosis can rapidly improve patient outcomes (4). However, many recommended antibiotics are expensive and increasingly ineffective due to bacterial resistance, making them difficult to implement with limited health services (4). Thus, shigellosis remains a persistent cause of mortality, particularly in young children, with insufficient practical options for disease management. Alternatives to expensive and logistically challenging antibiotic treatments for shigellosis could valuably reduce disease burden, especially in children.

As rates of antibiotic resistance in bacteria continue to increase alarmingly, complementary treatments to control bacterial infections are quickly being considered. One such treatment is phage therapy: the clinical use of bacteriophages (phages), viruses that specifically infect bacteria, to treat bacterial infections (5). Lytic phages are considered particularly good candidates for phage therapy due to their predator-like effect on susceptible host bacteria. The life cycle of a lytic phage involves attachment to one or more receptors on the surface of a bacterium, introduction of genomic material to the cytoplasm, intracellular replication, transcription and translation of phage genes, assembly of new phage particles, and lysis of the bacterial host to repeat the cycle (5). There are recent reports of implementation of phage therapy to treat refractory bacterial infections, particularly those that are resistant to multiple antibiotics (6–8). While phage therapy has demonstrated potential and promise as a complementary therapy to antibiotics, it is expected that these viruses will select for the evolution of phage-resistance in target bacteria. Therefore, it is crucial to design rational phage treatments, so that the evolution of phage resistance is leveraged as a possible clinical benefit, rather than necessarily constituting a barrier to effective treatment.

The concept of evolutionary trade-offs permeates evolutionary biology. Trade-offs occur when an organism evolves a new phenotype that improves fitness in a certain environment at the cost of decreased fitness in another environment (9). In the context of phage therapy, it should be possible to identify phages that direct the evolution of their host bacteria, such that the evolution of phage resistance (on average) results in a clinically useful fitness trade-off. In particular, choosing a lytic phage which exerts selection pressure on an antibiotic-resistance and/or virulence mechanism kills phage-susceptible bacteria while the remaining population is enriched for phage-resistant variants with reduced drug resistance and/or virulence (10, 11). In either case, the phage treatment could leverage evolution of phage resistance as a benefit rather than a strict liability.

To implement a rationally designed phage treatment that can select for a virulence trade-off in bacteria, we must identify a surface-expressed virulence factor that the phage uses for binding. Intercellular spread of S. flexneri contributes greatly to tissue damage and its accompanying symptoms, while providing the bacteria refuge from immune system detection and from antibiotics that transit poorly across eukaryotic cell membranes. One of the plasmid-encoded virulence factors of S. flexneri, IcsA, recruits and polymerizes actin at one end of the bacterial cell to create actin tails (12). These actin tails move the bacterium around the cytoplasm of a eukaryotic host cell, occasionally propelling the bacterium into the membrane and causing protrusions which can lead to the spread of the bacterium to a neighboring host cell (13). IcsA is both surface expressed and essential for S. flexneri virulence, suggesting its potential usefulness as a binding receptor for a phage therapy candidate. However, IcsA is plasmid encoded (14), indicating that this virulence factor might be spontaneously lost from a target bacteria during binary fission, or capable of horizontally transferring into cells of nontarget bacteria. For this reason, a better strategy would be to discover a phage that interacts with a known chromosomally-encoded virulence factor of S. flexneri bacteria, rather than targeting a genetic element that could be more easily gained or lost. Recent reports have implicated other outer membrane proteins (15, 16) as being necessary for the intercellular spread of S. flexneri, including OmpA, a highly conserved outer membrane porin; that is, a pore or channel in Gram-negative bacteria that translocates small molecules across the membrane. In S. flexneri, OmpA is surface expressed, required for virulence, and chromosomally encoded; these three properties, in addition to examples of other phages that require OmpA as a receptor (17), make it an attractive target for testing our hypothesis due to its potential as a phage receptor which could be leveraged to select against virulence in this pathogen.

In this study, we describe the recently isolated lytic phage A1-1, a double-stranded DNA (dsDNA) virus discovered in a wastewater sample obtained in Cuatro Cienegas, Mexico, a geographic region renowned for its extreme microbial diversity (18). Characterization of phage A1-1 revealed that it is a member of the *Myoviridae* family, and that the virus naturally uses OmpA as a binding receptor to infect susceptible S. flexneri bacterial cells. We hypothesized that phage A1-1would kill its host bacteria while selecting for evolution of resistance in the bacterial population, potentially causing a trade-off in the form of decreased virulence (i.e., reduced capacity for phage-resistant bacterial mutants to undergo intercellular spread). We randomly isolated five spontaneous phage-resistant mutants, and our phenotypic and genotypic characterizations revealed that these strains could be generally placed into two distinct groups: OmpA-deficient mutants and LPS-deficient mutants. Nevertheless, our data confirmed that all the isolated mutants suffered the predicted trade-off: these bacteria could invade and replicate in mammalian-derived cells in a tissue culture model of S. flexneri infection, but the evolved phage resistance was always associated with attenuated virulence because the mutants were incapable of intercellular spread.

## RESULTS

### Novel lytic phage A1-1 is a *Myoviridae* virus that binds to OmpA of *Shigella flexneri*.

We predicted that naturally-occurring lytic phages of S. flexneri have evolved to exploit OmpA as a receptor. We sought to isolate such a phage from water samples, assuming that these contained high levels of microbial biodiversity. Phage A1-1 was isolated from wastewater in Cuatro Cienegas, Mexico, purified on an avirulent strain of S. flexneri, PE577, using classical phage-isolation techniques, and then grown using S. flexneri strain M90T as a host in the experiments below.

**FIG 1:**
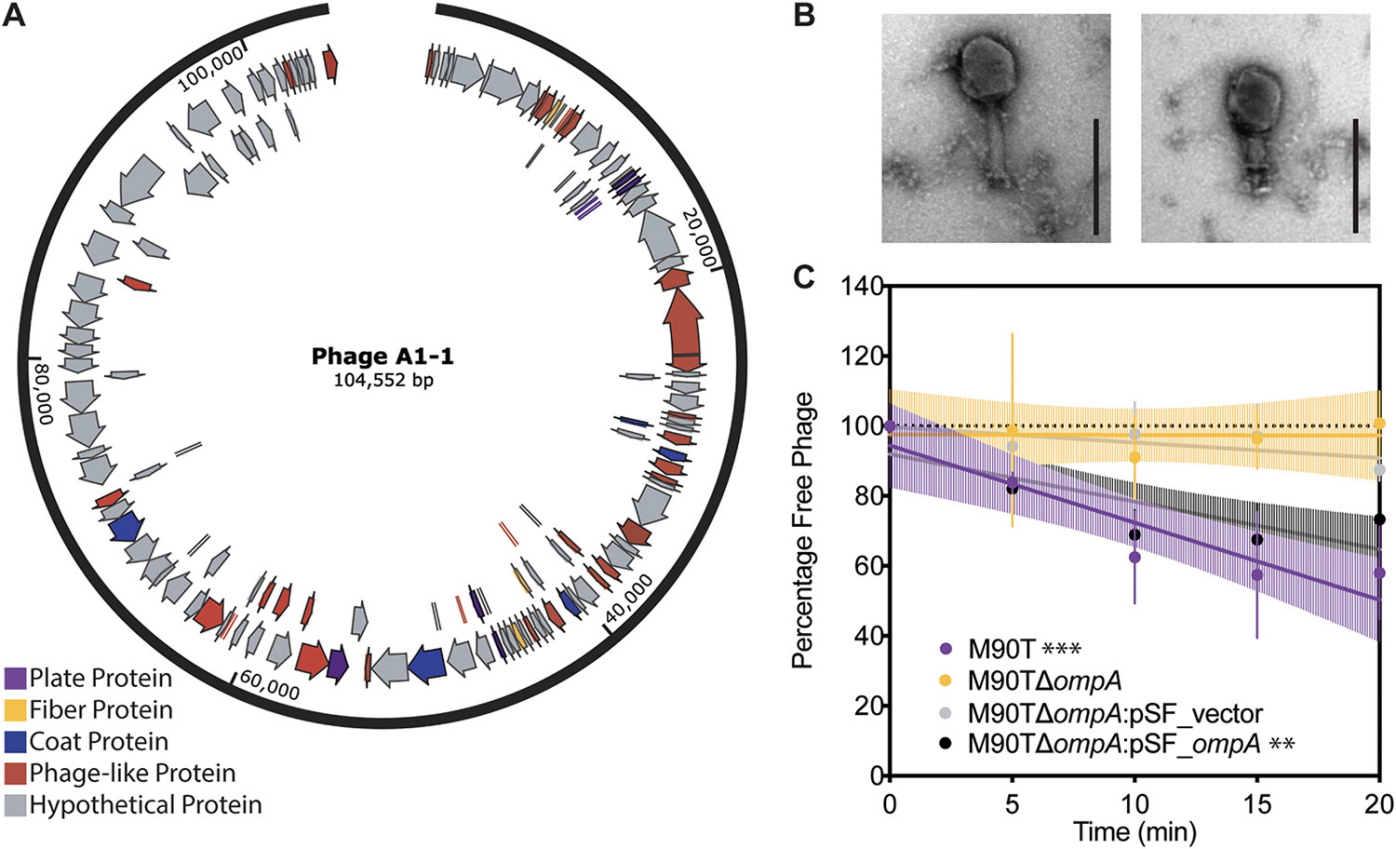
Basic characterization of newly discovered lytic phage A1-1 on host, Shigella flexneri. (A) Genome annotation of phage A1-1 using PHASTER. Plate proteins are shown in purple, fiber proteins in yellow, coat proteins in blue, phage-like proteins in red and hypothetical proteins in gray. (B) TEM image of phage A1-1 (200-nm scale bar). (C) Adsorption assay of phage A1-1 on bacterial hosts: M90T (purple), M90TΔ*ompA* (yellow), M90TΔ*ompA*:pSF_vector (gray), and M90TΔ*ompA*:pSF_*ompA* (black). Error bars show standard deviations of the mean for three biological replicates. Significance determined by testing whether slope of the linear regression line deviated from zero. (*, *P* < 0.05; **, *P* < 0.01; ***, *P* < 0.001).

We conducted whole-genome sequencing and used SPAdes (19) for *de novo* assembly of the phage A1-1 genome. Results showed an estimated genome size of 104,552 bp. Phage A1-1 was observed to have a GC content of 35.89%, whereas M90T had a GC content of 50.9% (20). Preliminary annotation of phage genes using PHASTER (21) revealed 139 coding regions (Fig. 1A) with no indication that the genes for lysogeny (temperate phage life cycle) were present. Sequence alignment suggested that phage A1-1 was similar to myoviruses known to infect Escherichia coli. Transmission electron microscopy (TEM) revealed that phage A1-1 had a long contractile tail (Fig. 1B); this morphology is consistent with the typical structure of virus particles of the *Myoviridae* family of dsDNA viruses.

We then conducted phage growth curves, in triplicate, which estimated key features of the phage A1-1 lytic reproductive cycle, and tested its ability versus inability to infect wild-type and OmpA knockout genotypes of S. flexneri strain M90T, respectively. Results showed that phage A1-1 had a latent and eclipse period of 33.33 ± 5.77 min (mean ± standard deviation [SD]), and a burst size of roughly 16.24 ± 12.39 phage particles (mean ± SD) (see Fig. S1A in the supplemental material). Furthermore, these data confirmed that phage A1-1 was able to grow on M90T but unable to infect M90TΔ*ompA* which lacked the putative OmpA binding site.

To determine conclusively whether phage A1-1 required OmpA to bind and infect S. flexneri cells, we performed adsorption assays to measure cell binding and efficiency of plaquing (EOP) assays to measure infectivity of phage A1-1. The results of replicated (*n *= 3) adsorption assays showed that phage A1-1 was unable to adsorb to both M90TΔ*ompA* and M90TΔ*ompA*:pSF-vector (empty vector control), with linear regression slopes of −0.016 ± 0.49 and −0.44 ± 0.23 (mean ± standard error of the mean [SEM]), respectively, which were not statistically significantly different from zero (Fig. 1C). However, we observed that phage A1-1 was able to adsorb to wild-type M90T as well as to M90TΔ*ompA*:pSF-*ompA*, with linear regression slopes of −2.21 ± 0.45 and −1.36 ± 0.35 (mean ± SEM), respectively (Fig. 1C). Similarly, EOP assays performed in triplicate (Fig. S1B) showed that phage A1-1 was able to infect M90TΔ*ompA*:pSF-*ompA* with an EOP of 0.365 ± 0.15 (mean ± SEM), but unable to infect M90TΔ*ompA* or M90TΔ*ompA*:pSF-vector with EOPs below the limit of detection (200 PFU/ml) on both strains. Finally, we examined growth kinetics of S. flexneri in the presence of phage A1-1 using bacterial growth curves. As expected, growth of the susceptible wild-type strain, M90T, was completely suppressed in the presence of phage A1-1 over the 15-h assay (Fig. S1C). Conversely, the growth of M90TΔ*ompA* and M90TΔ*ompA*:pSF-vector strains were both unimpacted by phage A1-1 (Fig. S1C). While growth of M90TΔ*ompA*:pSF-*ompA* was suppressed by phage presence, this reduced growth was less severe compared to that of the M90T wild type, likely due to greater chromosomal expression versus exogeneous expression of OmpA (Fig. S1C). Taken together, the above results showed that phage A1-1 was a naturally occurring virus that could be isolated and characterized as requiring OmpA to bind and initiate infection of S. flexneri host bacteria.

### Phage A1-1 selects for two different phenotypes of phage-resistant mutants.

Since the above results showed that phage A1-1 used OmpA to infect S. flexneri, we hypothesized that the evolution of phage resistance could occur via modification of the phage receptor, OmpA, and that phage-resistant mutants would be phenotypically similar to M90TΔ*ompA*. Fluctuation assays revealed that phage A1-1 selected for phage-resistant mutants of M90T at a frequency of 4.18 × 10^−7^ ± 1.22 × 10^−7^ (mean ± SD). We randomly chose five independently-isolated spontaneous phage-resistant mutants (here, R1, R2, R3, R4, and R5) for further characterization.

**FIG 2:**
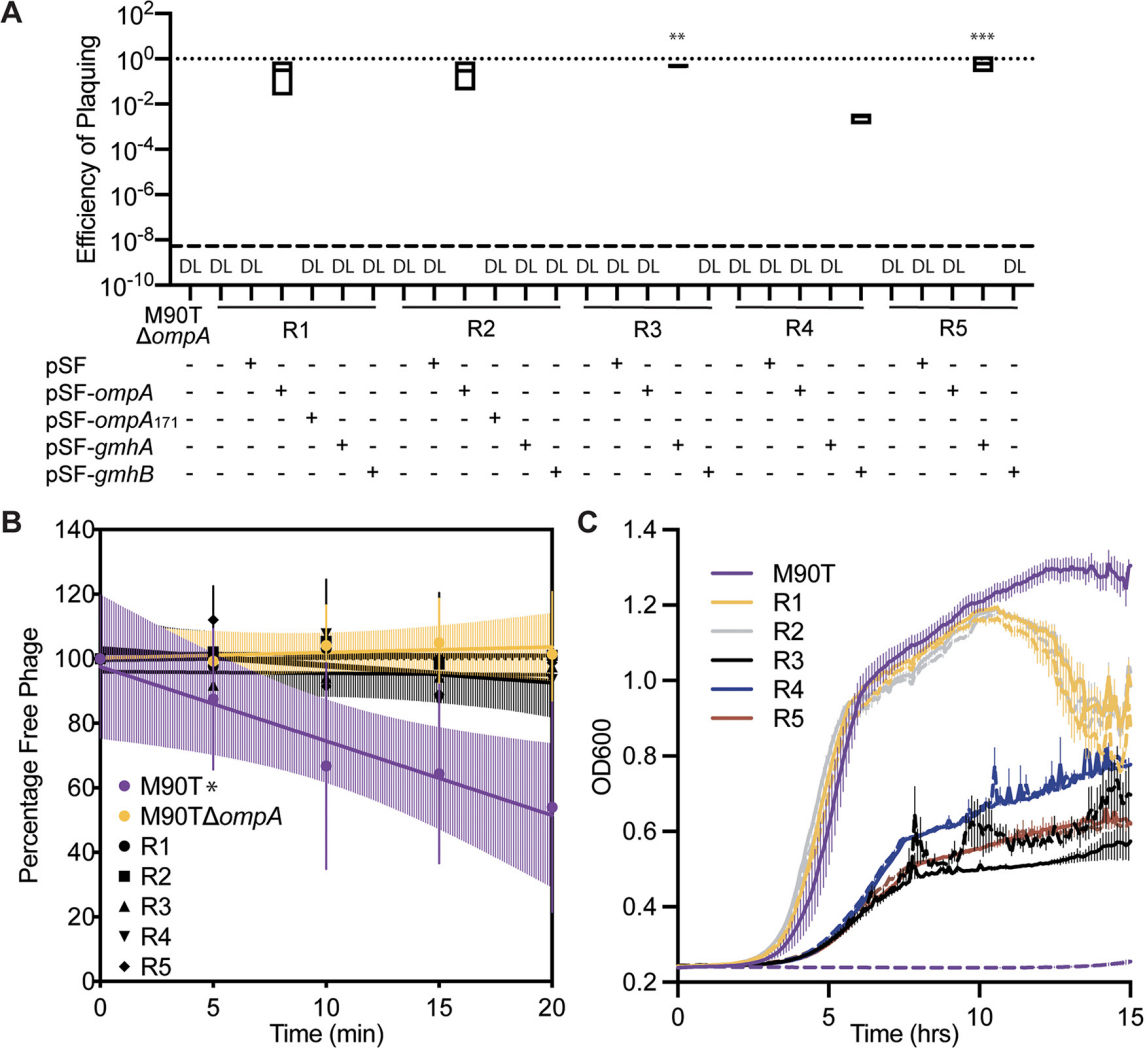
Characterization of phage resistance. (A) Efficiency of plaquing (EOP) assay of phage A1-1 on various strains. Dotted line at 1 indicates EOP on M90T. “DL” indicates that the EOP was below the limit of detection (dashed line). Significance determined by a one-way ANOVA followed by Dunnet’s multiple-comparison test to M90TΔ*ompA*. (B) Adsorption assay of phage A1-1 on bacterial hosts: M90T, M90TΔ*ompA*, R1, R2, R3, R4, and R5. Error bars show standard deviations of the mean of three biological replicates. Significance determined by testing whether slope of the linear regression line deviated from zero. (C) Growth curves of M90T (purple), R1 (yellow), R2 (gray), R3 (black) R4 (blue), and R5 (red). Solid lines indicate bacteria growing in the absence of phage; dashed lines indicate phage A1-1 presence (MOI = 10). (*, *P* < 0.05; **, *P* < 0.01; ***, *P* < 0.001).

Using similar assays as above, we sought to confirm that all five mutants showed traits associated with phage resistance. As expected, phage A1-1 had an EOP below the limit of detection (200 PFU/mL) on each resistant mutant (Fig. 2A). Similar to data for M90TΔ*ompA* presented above, all five resistant mutants did not support phage adsorption, as indicated by the slopes of linear regressions that were not statistically significantly different from zero (Fig. 2B). Also, growth of the phage-resistant mutants was not visibly altered by the presence of phage A1-1, while growth of wild-type M90T was completely suppressed by phage A1-1 (Fig. 2C). We noted that in the absence of phages, mutants R1 and R2 grew to final densities similar to that of the M90T ancestor, whereas mutants R3, R4, and R5 reached final densities that were roughly 2-fold less than the that of the wild type (Fig. 2C). Furthermore, R1 and R2 produced phenotypically “smooth” colonies when grown on agar, while R3, R4, and R5 grew as “rough” colonies on agar, indicating that the latter mutants had potentially been altered for lipopolysaccharide (LPS) production. In summary, these results confirmed that all the mutants had phenotypic traits consistent with resistance to phage A1-1 infection, but the strains could be separated into two groups: R1 and R2; and R3, R4, and R5.

**FIG 3:**
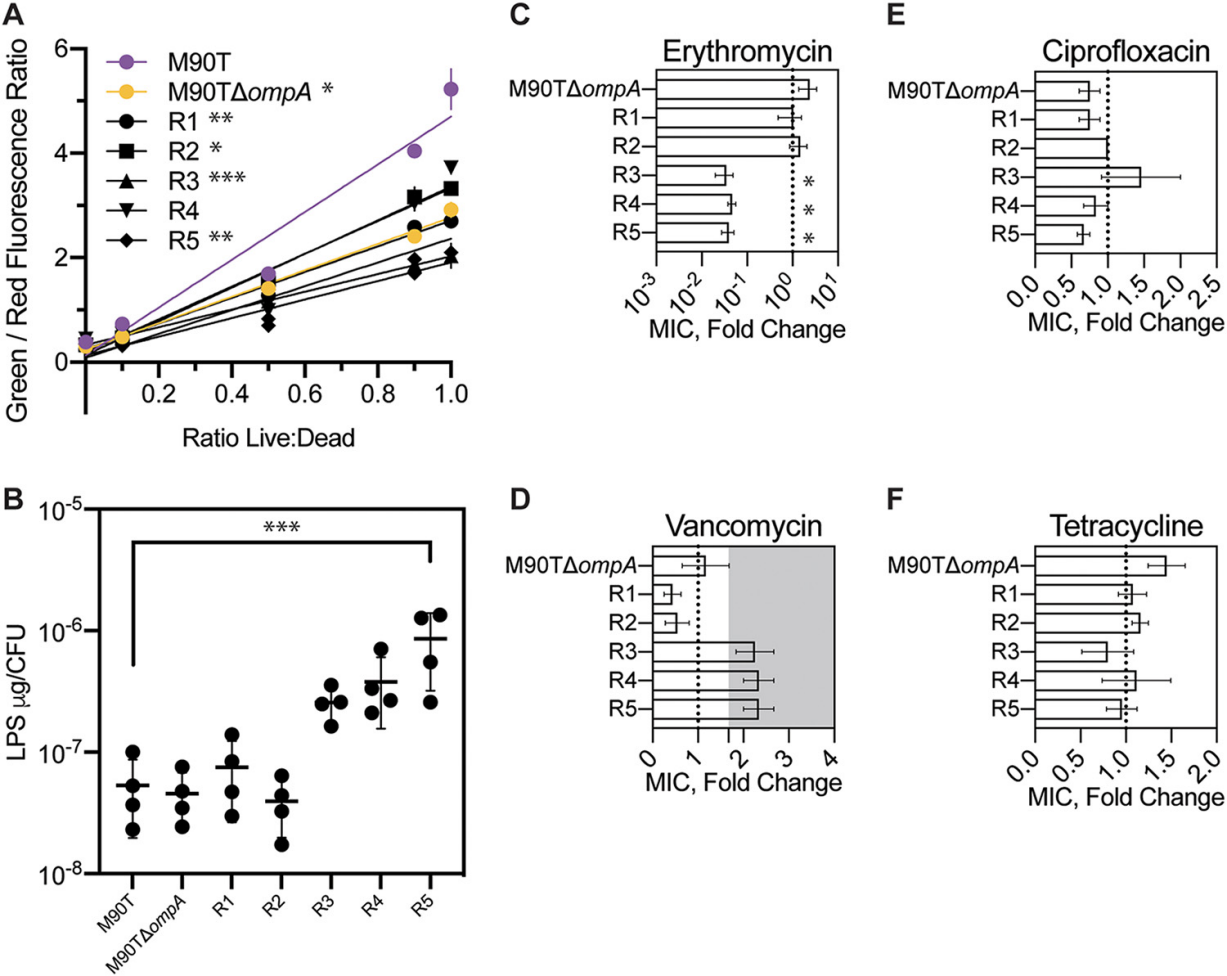
Phenotypic characterization of five spontaneous phage-resistant mutants of S. flexneri. (A) Membrane permeability measured as a linear regression of the ratio of green to red fluorescence for different suspensions of live and dead cells stained with SYTO 9 dye and propidium iodide. Error bars show standard deviations of the mean of two technical replicates, and data are representative of 3 separate experiments analyzed by one-way ANOVA of the slopes of linear regressions using Dunnet’s multiple comparisons to M90T. (B) Total LPS measured as μg per CFU. Error bars show standard deviations of the mean of four biological replicates (one-way ANOVA with Dunnet’s multiple comparisons to M90T). (C through F) Fold change in MIC compared to MIC of wild-type M90T for erythromycin, vancomycin (shaded region indicates limit of detection), ciprofloxacin, and tetracycline. Error bars show standard errors of the mean for three to four biological replicates (one-way ANOVA with Dunnet’s multiple comparisons to M90TΔ*ompA*). (*, *P* < 0.05; **, *P* < 0.01; ***, *P* < 0.001).

We used assays that investigated membrane permeability, LPS quantity per cell, and resistance to four antibiotics, anticipating changes (relative to wild type) in these traits if phage resistance had been conferred by altering OmpA and/or LPS to further characterize the five phage-resistant mutants. Membrane-integrity assays were used as proxies for cell permeability, and the results showed that the permeabilities of M90TΔ*ompA* and resistant mutants R1, R2, R3, and R5 were statistically significantly lower than that of the M90T wild-type (Fig. 3A). Total LPS measurements revealed that while R1 and R2 had similar amounts of LPS per CFU compared to M90T and M90TΔ*ompA*, R3, R4, and R5 showed almost an order of magnitude more LPS per CFU (Fig. 3B). We expected that alterations to OmpA and LPS would result in changes in MIC for certain antibiotics. Mutants R3, R4, and R5 showed a significant decrease in mean fold change (compared to the wild type) in the MIC of erythromycin (R3, 0.042 ± 0.015; R4, 0.056 ± 0.009; R5, 0.071 ± 0.01; mean ± SEM.) (Fig. 3C). Mutants R1 and R2 showed a slightly decreased fold change in vancomycin MIC, while mutants R3, R4, and R5 had slightly increased fold changes in vancomycin MICs (Fig. 3D). Fold change in the MICs of ciprofloxacin and tetracycline was similar to that of the wild type for all five mutants (Fig. 3E and F). We further concluded that phage A1-1 tended to select for phage-resistant mutants in two groups: R1 and R2 likely had mutations affecting OmpA, while R3, R4, and R5 likely had mutations altering LPS.

To further investigate the potential role of LPS in phage resistance, EOPs were measured on E. coli knockout strains (22) for genes involved in LPS biosynthesis (23). The EOP of phage A1-1 was below the limit of detection when the following 15 E. coli genes were knocked out: *ompA*, *galU*, *waaC*, *gmhA*, *gmhB*, *gmhC*, *gmhD*, *waaF*, *waaQ*, *waaP*, *waaY*, *waaG*, *waaO*, *waaJ*, and *waaB* (Fig. S2). The EOP measurements for phage A1-1 on the BW25113Δ*icdC* and BW25113Δ*ompC* controls, as well as on the BW25113Δ*waaS* and BW25113Δ*waaL* knockouts, were not statistically different from phage ability to grow on wild-type BW25113 (Fig. S2). In contrast, the EOP of phage A1-1 on BW25113Δ*waaZ* was slightly improved compared to that of the wild type BW25113 (Fig. S2). These results suggested that the LPS biosynthesis genes *waaS*, *waaL*, and *waaZ* were not determinants of phage A1-1 growth. From these results, we concluded that portions of S. flexneri LPS were somehow required for phage A1-1 infection.

Since our earlier results showed that phage A1-1 used OmpA to bind and initiate infection of S. flexneri, we anticipated that the genetics underlying phage resistance should be governed by alterations of OmpA and not by changes in LPS. While the previous results (Fig. 1) hinted that OmpA might be the only receptor for phage A1-1, EOP data from the E. coli LPS knockout strains implied that further tests were needed to examine whether LPS also served as a receptor. To that end, we tested whether resistance to phage A1-1 additionally altered susceptibilities of the resistant mutants to the previously characterized phages 60B and T7. Phage 60B putatively requires OmpC as a receptor. Our results showed that wild-type M90T and the mutants R1 and R2 were susceptible to phage 60B infection, indicating that this constituted the ancestral phenotype, whereas our data showed that mutants R3, R4, and R5 displayed resistance to phage 60B (Table S1). E. coli phage T7 uses the inner core of LPS as a receptor (24). Results showed that wild-type M90T and mutants R1 and R2 were resistant to phage T7, indicating that T7 resistance was the ancestral phenotype, while R3, R4 and R5 were susceptible to phage T7 (Table S1). Differences in phage T7 resistance again suggested that putative LPS changes conferred phage A1-1 resistance in mutants R3, R4, and R5. Moreover, the differences in phage 60B resistance for these strains compared to the wild type suggested that LPS changes may broadly affect the structures of different outer membrane porins. Therefore, we hypothesized that while LPS might be necessary for A1-1 infection, it was not serving as a receptor; rather, LPS could serve to maintain the appropriate structure of the phage A1-1 binding receptor OmpA.

**FIG 4:**
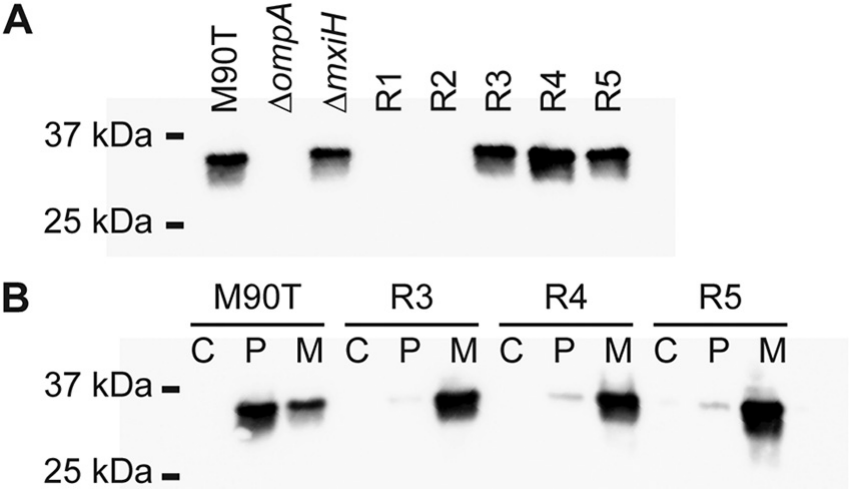
OmpA expression in phage-resistant mutants of S. flexneri, compared to controls. (A) Western blot for OmpA of whole bacterial cell extract of M90T, M90TΔ*ompA*, M90TΔ*mxiH*, R1, R2, R3, R4, and R5. (B) Western blot for OmpA of cytoplasmic (C), periplasmic (P) and membrane (M) fractions of M90T, R3, R4 and R5.

Western blotting was used to determine whether the five resistant mutants expressed OmpA. Blots for OmpA of whole-cell lysates showed that resistant mutants R1 and R2 did not express OmpA; however, R3, R4, and R5 expressed OmpA to a similar degree as the wild-type control (Fig. 4A). Furthermore, Western blots of fractioned cell lysates revealed that R3, R4, and R5 expressed OmpA at the membrane (Fig. 4B). These results supported our hypothesis that LPS was probably not a receptor for phage A1-1, but instead maintained a conformation of OmpA that was required for phage A1-1 binding.

Based on differing results of observed colony morphologies, MICs of various antibiotics, phage A1-1 infection ability on LPS mutants of E. coli, and Western blots for OmpA, we concluded that phage-resistant mutants R1 and R2 phenotypically resembled the M90TΔ*ompA* knockout and did not express this OmpA, while R3, R4, and R5 appeared to be phage A1-1 resistant due to changes in LPS structure.

### Whole-genome sequencing reveals underlying mutations conferring resistance.

**FIG 5:**
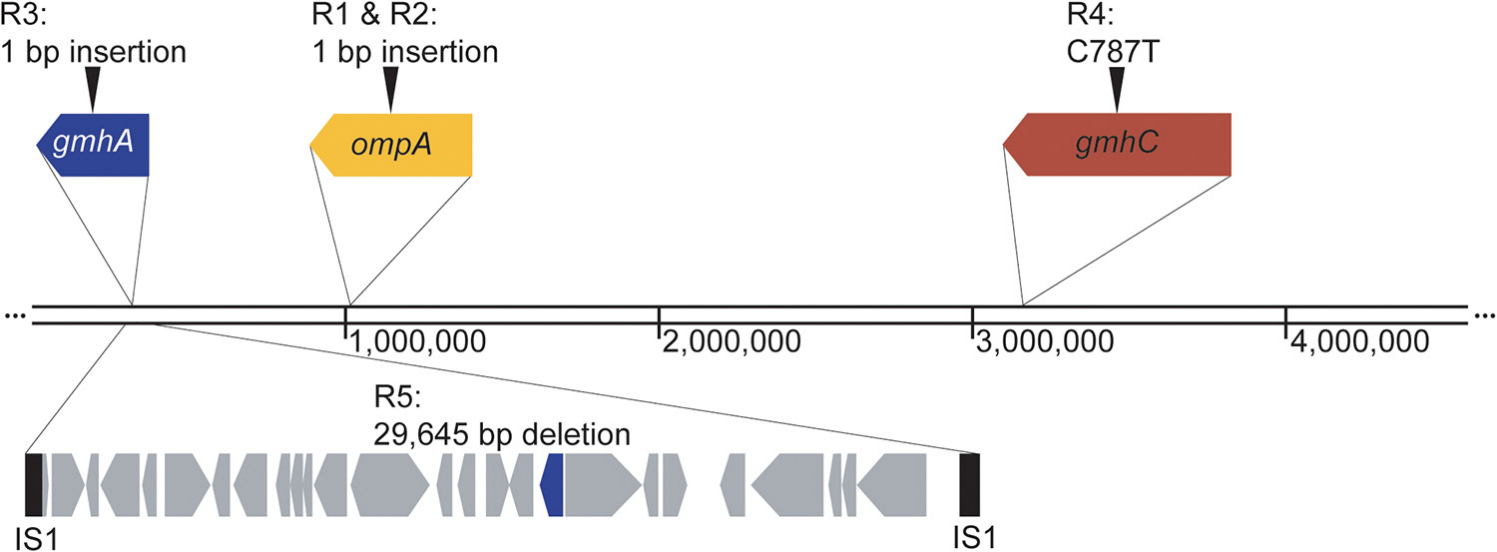
Diagram of mutations identified via whole-genome sequencing. The chromosome of wild-type M90T is 4,580,866 bp. Mutations were identified in all five phage-resistant mutants. R1 and R2 have identical single base pair insertions at position 507 in *ompA* (yellow). R3 has a single base pair insertion in *gmhA* (blue) at position 400. R4 has a transversion at position 787 of *gmhC* (red). R5 has a 29,645-bp deletion spanning 27 genes, including *gmhA* (blue), between two IS1 elements (black).

Whole-genome sequencing was used to determine whether the genotypes of the phage-resistant bacterial mutants matched our inferences based on the above-described phenotypes. Genomes of all five resistant mutants were sequenced, aligned to the reference genome for the wild-type M90T ancestor (GenBank no. CM001474.1), and GATK (25) was used to identify variants. Results showed that both R1 and R2 had a nonsense mutation in *ompA*, resulting in a premature stop codon after amino acid 172 (Fig. 5). R3 had a nonsense mutation in the LPS biosynthesis gene *gmhA*, which resulted in a premature stop codon after amino acid 176 (Fig. 5). R4 had a transversion at base pair 787 of the LPS biosynthesis gene *gmhC*. Finally, R5 had a 29,645-bp deletion between two insertion sequence 1 (IS1) elements at positions 303,277 and 332,873; this deletion included 27 genes, one of which was *gmhA* (Fig. 5). The EOP of phage A1-1 was restored to the levels observed with wild-type *S. flexneri* upon complementation of R1 and R2 with pSF-*ompA*, R3 and R5 with pSF-*gmhA*, and R4 with pSF-*gmhC* (Fig. 2A). Altogether, whole-genome sequencing and EOP assays confirmed that R1 and R2 were *ompA* mutants, while R3, R4, and R5 were LPS-deficient mutants.

### Phage-resistant mutants are attenuated for intercellular spread.

We originally sought to test whether a phage that required OmpA to bind and infect S. flexneri would select for phage-resistant mutants that were attenuated for intercellular spread.

**FIG 6:**
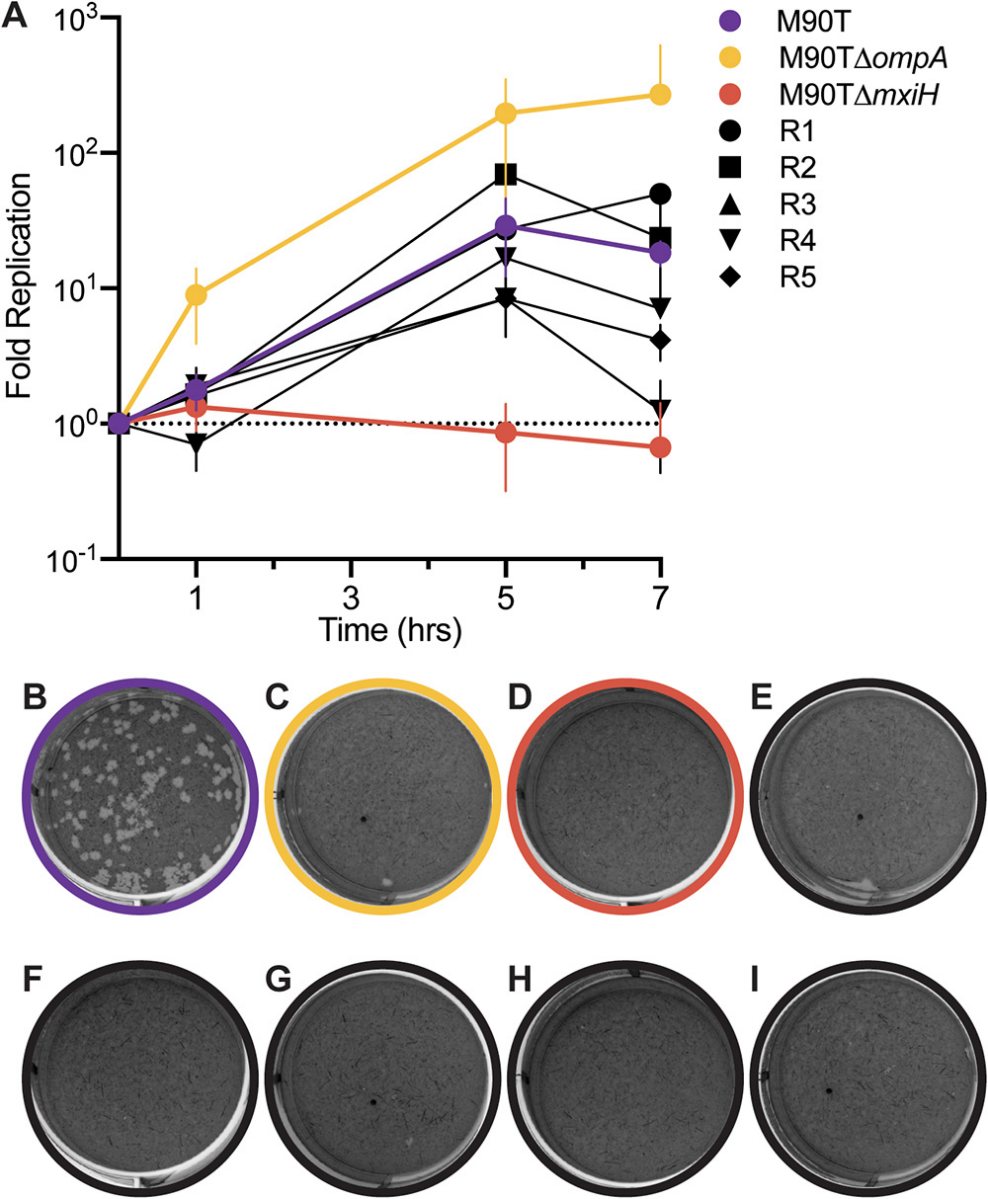
Virulence of phage-resistant mutants, relative to controls, in a tissue-culture model of S. flexneri pathogenicity. (A) Intracellular growth curves of wild-type M90T (purple), M90TΔ*ompA* (yellow), M90TΔ*mxiH* (red), R1 (black circle), R2 (black square), R3 (black triangle), R4 (black inverted triangle), and R5 (black diamond) bacteria. Error bars show standard deviations of the mean of three technical replicates. (B through I) Plaque assays of wild-type M90T, M90TΔ*ompA*, M90TΔ*mxiH*, R1, R2, R3, R4, and R5, respectively, on monolayers of Vero cells at MOI of 5 (bacteria CFU relative to Vero cells) after 72 h incubation. Data are representative of at least three separate experiments.

We first compared the abilities of the phage-resistant mutants to enter and replicate within eukaryotic cells, using intracellular replication assays and plaque assays in primate-derived Vero cells grown in laboratory tissue culture. As expected, M90T and M90TΔ*ompA* bacteria were capable of invading and replicating in Vero cells, while the negative control M90TΔ*mxiH*, a knockout for the type III secretion system needle, was unable to invade (Fig. 6A). All five of the phage-resistant mutants were able to invade and replicate intracellularly; R1, R2, R3, and R5 replicated to levels similar to that of M90T at the 7-h time point, while R4 replicated 10-fold less than M90T by this time (Fig. 6A).

We conducted plaque assays on Vero cells to measure whether the bacteria could undergo cell-to-cell spread, and results confirmed that wild-type M90T (Fig. 6B) could spread intercellularly. In contrast, the data revealed that neither M90TΔ*ompA* nor M90TΔ*mxiH* (Fig. 6B and C) could spread intercellularly, and that none of the five phage-resistant mutants (Fig. 6D to I) could spread between cells. Therefore, as predicted, although all five resistant mutants were able to invade and replicate intracellularly, they were unable to spread intercellularly, indicating that the evolution of resistance to phage A1-1 led to generalized reduction of virulence in S. flexneri.

## DISCUSSION

Classical phage biology assays, including adsorption assays, EOPs, and growth curves, suggest that phage A1-1 uses OmpA as a receptor in S. flexneri. Phage A1-1 selected for two different phenotypes of phage-resistant mutants. R1 and R2 formed smooth colonies and exhibited decreased membrane permeability and increased vancomycin sensitivity compared to the wild type. R3, R4, and R5 formed rough colonies and exhibited decreased membrane permeability, increased erythromycin sensitivity, increased vancomycin resistance, and greater total LPS per CFU when compared to the wild-type; they also showed altered phage resistance profiles to phages 60B and T7. Whole-genome sequencing revealed that the phage selected for resistant bacteria with mutations in *ompA* (R1 and R2). In addition, we observed that phage A1-1 selected for resistant bacteria with mutations in *gmhA* (R3 and R5) and *gmhC* (R4), two genes involved in the biosynthesis of the heptose sugars that make up the inner core of LPS (26). Resistant mutants R3, R4, and R5 all express OmpA on the membrane, and phage A1-1 fails to adsorb to these resistant mutants. Altogether, these observations suggest two possibilities: either phage A1-1 uses both OmpA and LPS as coreceptors, or phage A1-1 uses OmpA as a receptor and LPS is somehow required for binding without serving as a receptor.

Our data strongly suggest that phage A1-1 uses OmpA as a receptor, and that certain mutations in LPS genes can reduce phage binding due to interactions between OmpA and LPS. We noted that plaques of phage A1-1 were never observed on M90TΔ*ompA*, even when high titers of the virus were plated on M90TΔ*ompA*. If both OmpA and LPS were coreceptors for phage A1-1, we would expect that spontaneous mutation in the phage population (i.e., standing genetic variation) combined with strong selection for host use should permit rare variants of phage A1-1 to plaque on M90TΔ*ompA*, using only LPS as a receptor. However, this was not observed. Instead, it seems likely that A1-1 uses OmpA as a primary receptor and that LPS is required for binding either as a secondary receptor or to maintain the proper conformation of OmpA for A1-1 binding. Since resistant mutants R3, R4, and R5 all express OmpA and no phage adsorption to these resistant mutants was observed, it seems unlikely that LPS is being used as a secondary receptor by phage A1-1. Therefore, perhaps LPS is involved in maintaining a particular conformation of OmpA that is necessary for phage binding. Indeed, it has been previously observed that complex interactions between LPS and outer membrane proteins (OMPs) are involved in both OMP biogenesis and the stabilization of particular conformations of various OMPs (27–29). Three different conformations of E. coli OmpA have been proposed: a monomeric narrow-pore conformation with two domains (an eight-stranded β-barrel and a periplasmic domain), a monomeric large-pore conformation with a single domain (16-stranded β-barrel), and a dimer of the two-domain narrow-pore structure (30–32). OmpA of M90T appears to be localized to both the periplasm and membrane fractions (Fig. 3B), indicating that perhaps the two-domain narrow-pore structure with the periplasmic domain is the main conformation of OmpA in these bacteria. Interestingly, OmpA in the resistant mutants R3, R4, and R5 is localized more in the membrane fraction than in the periplasmic fraction (Fig. 3B), possibly indicating that the single-domain large-pore structure dominates the conformation state of OmpA in these LPS-deficient mutants. Indeed, it has been previously suggested that core LPS plays a role in maintaining the conformation of OmpA (33). Experiments in the current study seem to support this hypothesis.

When S. flexneri invades and replicates within human host cells, it is known to spread intercellularly via actin-based motility, which occurs through the polymerization of host actin by unipolar IcsA on the bacteria. It has previously been demonstrated that O-antigen null mutants (i.e., those with truncated LPS) are attenuated for intercellular spread (34). Therefore, it is perhaps unsurprising that the phage-resistant LPS mutants R3, R4, and R5 are capable of intracellular invasion and replication but unable to spread from cell to cell. However, the mechanism by which O-antigen confers the ability for S. flexneri to spread intercellularly has not been fully elucidated. Furthermore, prior work shows that OmpA is required for intercellular spread (15); as expected, our isolated phage-resistant mutants with OmpA changes, R1 and R2, are similarly defective for this virulence trait. Interestingly, the shared *ompA* mutation that purportedly confers phage resistance in R1 and R2 is in the region that likely composes the final predicted extracellular loop of the eight-stranded β-barrel conformation and truncates OmpA at position 174. It has been observed that OmpA residues 188 to 190 interact with a periplasmic protein, PhoN2, to maintain the polarity of IcsA (35). Therefore, if these two resistant mutants express a partial peptide of OmpA, where residues 1 to 169 are wild type and 170 to 173 are frameshifted, it is likely that this partial protein is not suitable for phage A1-1 adsorption nor has the region predicted to interact with PhoN2 to maintain polarity of IcsA. This perhaps explains the observed defect in intercellular spread observed for phage-resistant mutants R1 and R2.

It is unsurprising that *ompA* and LPS biosynthesis mutants of S. flexneri in the current study are attenuated for intercellular spread, based on previous literature, but it is noteworthy that these mutants were obtained strictly via selection for resistance to phage A1-1. In all five randomly chosen mutants, phage resistance led to attenuation of intercellular spread; thus, selection for resistance to phage A1-1 in S. flexneri consistently traded off with the maintenance of a key virulence factor in this biomedically important pathogen. While this interesting result yields insights into possible S. flexneri interactions with lytic phages, expectations for phage-bacteria interactions in natural environments and in the context of clinical infection are harder to predict (36). The epidemiology of S. flexneri typically involves the colonization of an index human case (presumably via consumption of contaminated food or water) which can then be fecal-orally transmitted to household members and other nearby individuals. As a facultative intracellular pathogen, S. flexneri is capable of growth inside human host cells but can also exist as a “free-living” bacterium in the natural environment. During clinical infections, bacteria are presumably protected from phage attack while replicating inside colonic epithelial cells; however, during fecal-oral transmission, bacteria are free-living and once again vulnerable to phage. This is an example of an alternating environment wherein bacteria experience intermittently changing selection pressures. That is, aside from other possible selection pressures such as escape from host immunity, a population of S. flexneri may sometimes experience selection to avoid phage infection, and other times experience the absence of this selection if intracellular invasion provides a refuge from phage exposure. Under such complex conditions, selection would positively favor evolution of phage resistance in free-living bacteria; in this ecology, the mutations underlying phage resistance would be beneficial and positively selected. However, inside intestinal epithelial cells, the ecology presents a different target for selection; here, the mutations for phage resistance observed in this study would be deleterious because of the trade-off we observed between phage resistance and intercellular spread.

This scenario stimulates two predictions: (i) the evolution of phage resistance (i.e., positive versus negative selection for this trait) should occur differently for facultative intracellular pathogens such as S. flexneri, depending whether the bacterial population is growing inside versus outside the human host; and (ii) the particular phage-resistant mutants in the current study should experience a net fitness cost if the frequency of environmental fluctuations necessitates intracellular growth and spread during infection of human host cells. The first prediction is the subject of ongoing studies in our laboratory to examine spontaneous phage-resistant mutants of S. flexneri selected under simultaneous selection pressures of phage A1-1 and intracellular growth constraints in Vero cells. The second prediction can be examined by subjecting the phage-resistant mutants in the current study to experimental evolution which toggles between batch culture and tissue culture environments. Here, the outcome can be informed by previous studies of microbial evolution in fluctuating environments. For example, it has been observed that phages subjected to elevated-temperature selection (i.e., heat shocks) outside of bacterial cells results in the evolution of greater thermotolerance (increased particle stability) to withstand degradation in elevated temperatures, despite the deleterious nature of these mutations for phage reproduction in benign temperature environments (37–39). Thus, antagonistically pleiotropic mutations can evolve in microbes, even though these changes confer high fitness in only a portion of the selective environment. We might expect that a fluctuating environment would cause the phage-resistant mutants in our study to undergo further evolution, such as compensation for their complete inability to spread intercellularly, to maintain some degree of phage resistance while also achieving minimal intercellular spread (a compromise). In contrast, our study may reveal that the evolution of phage resistance creates a strong evolutionary constraint whereby acquisition of phage A1-1 resistance prevents S. flexneri from readily undergoing compensatory evolution, effectively confining the bacteria to the free-living “ecology” when faced with selection to avoid phage attack. If this result holds true for the system presented in the current study, it might be possible to select for long-lasting phage-resistant S. flexneri strains that are attenuated for virulence, a key step toward developing phage treatments for this important human pathogen.

## MATERIALS AND METHODS

### Strains and culture conditions.

Bacteria, phage, and plasmid strains used in this study are listed in Tables 1 to 3. S. flexneri was grown in 0.1% Congo red-tryptic soy (CR-TS) medium (10 g tryptone, 5 g soytone, 10 g NaCl, and 0.1 g Congo red dye per L) or low-salt CR-TS (10 g tryptone, 5 g soytone, 5 g NaCl, and 0.1 g Congo red dye per L) unless otherwise noted. E. coli was grown in lysogeny broth (LB) medium (10 g tryptone, 5 g yeast extract, 10 g NaCl per L). Phage A1-1 was amplified to high titer on S. flexneri strain M90T unless otherwise noted. Bacteria were grown on 1.5% agar plates and phages were grown on plates with a 0.75% top agar layer. Plasmids were maintained using 100 μg/mL of carbenicillin and expression was induced with 1 mM isopropyl-β-d-thiogalactopyranoside. λ-Red recombination (40) was used to engineer bacterial strains M90TΔ*ompA*Kan^r^ and M90TΔ*mxiH*Kan^r^, and plasmid pCP20 was used to remove the Kan^r^ cassette from each of these strains to create strains M90TΔ*ompA* and M90TΔ*mxiH*.

**TABLE 1:** Bacterial and tissue culture strains used in this study^*a*^

Bacterial strain	Species	Antibiotic resistance cassette	Reference or source
PE577	S. flexneri	-	(17)
			
M90T	S. flexneri	SmR	ATCC BAA-2402
Δ*ompA* KanR	S. flexneri	SmR, KanR	This study
Δ*ompA*	S. flexneri	SmR	This study
Δ*mxiH* KanR	S. flexneri	SmR, KanR	This study
Δ*mxiH*	S. flexneri	SmR	This study
			
BW25113	E. coli	-	(22); CGSC
Δ*icdC*	E. coli	KanR	(22); CGSC
Δ*ompA*	E. coli	KanR	(22); CGSC
Δ*ompC*	E. coli	KanR	(22); CGSC
Δ*galU*	E. coli	KanR	(22); CGSC
Δ*waaC*	E. coli	KanR	(22); CGSC
Δ*gmhA*	E. coli	KanR	(22); CGSC
Δ*gmhB*	E. coli	KanR	(22); CGSC
Δ*gmhC*	E. coli	KanR	(22); CGSC
Δ*gmhD*	E. coli	KanR	(22); CGSC
Δ*waaF*	E. coli	KanR	(22); CGSC
Δ*waaQ*	E. coli	KanR	(22); CGSC
Δ*waaP*	E. coli	KanR	(22); CGSC
Δ*waaY*	E. coli	KanR	(22); CGSC
Δ*waaG*	E. coli	KanR	(22); CGSC
Δ*waaO*	E. coli	KanR	(22); CGSC
Δ*waaJ*	E. coli	KanR	(22); CGSC
Δ*waaB*	E. coli	KanR	(22); CGSC
Δ*waaS*	E. coli	KanR	(22); CGSC
Δ*waaL*	E. coli	KanR	(22); CGSC

a KanR, Kanamycin resistance; SmR, Streptomycin resistance; AmpR, Ampicillin resistance; CGSC, Coli Genetic Stock Center.

**TABLE 2:** Phage strains used in this study

Phage	Host species	Reference or source
A1-1	S. flexneri	This study
60B	S. flexneri	This study
T7	E. coli	J. Wertz (Yale U)

**TABLE 3:** Plasmid strains used in this study

Plasmid	Antibiotic resistance cassette	Source
pSF-vector	AmpR	Oxford Genetics (OGS634)
pSF-ompA	AmpR	This study

### Assays to characterize phage.

Adsorption assays to estimate phage cell-binding ability were performed as described by Kropinski (41). Efficiency-of-plating assays to estimate phage infectibility on challenge bacteria relative to that on a permissive host strain, and phage growth curves to estimate virus traits (i.e., latent period, exponential growth rate, burst size), were performed as previously described (42). Bacterial growth curves were either measured in the absence of phage infection or conducted at a multiplicity of infection (MOI; ratio of phage particles to bacterial cells) of approximately 10; representative growth curves from Fig. 2C and Fig. S1A are plotted individually in Fig. S3. Transmission electron microscopy (TEM) was performed using uranyl acetate-stained phages on 300-mesh carbon film copper grids, and phage particles were visualized on a FEI Tecnai Biotwin microscope.

### LPS extractions.

Lipopolysaccharide (LPS) was extracted from bacteria using an LPS extraction kit (Abcam ab239718) and quantified using a carbohydrate quantification assay (Abcam ab155891). LPS was extracted following the instructions provided in the kit. Briefly, bacteria were grown overnight on LB agar, scraped into 1 mL of phosphate-buffered saline (PBS), centrifuged for 5 min at 5,000 × *g* at 4°C, resuspended in extraction buffer, sonicated (3 times for 30 sec each, at 2 to 10 W, using a Q55 QSonica), and incubated on ice for 10 min to allow for full lysis. Any unlysed cells were removed via centrifugation (5 min at 5,000 × *g* at 4°C) and the supernatant was treated with proteinase K at 60°C for 1 h. Any remaining debris was removed via centrifugation (5 min at 5,000 × *g* at 4°C). Total carbohydrate content (grams/CFU) in the supernatant was quantified following the instructions for the carbohydrate quantification assay.

### MIC measurements.

MIC was measured using Etest strips (bioMérieux). Briefly, overnight cultures of bacteria were spread on Mueller-Hinton agar (MHA; 2 g beef extract, 17.5 g casein hydrolysate, 1.5 g starch, and 17 g agar per L) plates and test strips were plated. After overnight incubation, MIC values were determined by the lowest concentration at which bacterial growth was inhibited. These measurements were conducted with 3-fold replication.

### Membrane permeability assay.

Membrane permeability of cells grown in LB was assayed using the Live/Dead BacLight kit (ThermoFisher L34856) with a microplate reader (Tecan Infinite F500) following the manufacturer’s instructions.

### Cell fractionation, protein gels, and Western blotting.

Whole-cell lysates were made by centrifuging cells (5 min at 5,000 × *g*), resuspending in 1× Laemmli buffer (Bio-Rad) and boiling at 100°C for 15 min. Cell fractions were made following previously published protocols (43). Briefly, late log cultures were centrifuged at 5,000 × *g* for 5 min and gently resuspended in 200 μL of *N*-tris(hydroxymethyl)methyl-2-aminoethanesulfonic acid (TES) buffer (200 mM Tris-HCl [pH 8.0], 0.5 mM EDTA, 0.5 M sucrose). Lysozyme (final concentration 10 μg/mL) was added to the resuspended cells, followed by 720 μL of TES buffer that had been diluted 1:1 in water. The lysate was incubated for 30 min on ice with gentle inversion to mix, and then centrifuged at 5,000 × *g* for 5 min at 4°C. The supernatant was reserved as the periplasmic fraction. The spheroplast fraction (i.e., the pellet) was resuspended in TES buffer diluted 1:1 in water containing 2 mM phenylmethylsulfonyl fluoride (PMSF), 2 mM MgCl_2_ and 10 μg/mL of DNase I and lysed via four cycles of freeze-thaws from −273°C to 37°C. Unlysed spheroplasts were removed via centrifugation at 2,000 × *g* for 5 min at 4°C. Finally, the supernatant was ultracentrifuged at 120,000 × *g* for 45 min at 4°C. The supernatant was reserved as the cytoplasmic fraction, and the pellet was resuspended in TES buffer diluted 1:1 in water and saved as the membrane fraction. Whole-cell lysates and fractions were run at 200 V for 45 min on a 10% Tris-Glycine polyacrylamide gel and transferred to a PVDF membrane (90 V for 60 min) and blotted with an anti-OmpA antibody (Biorbyt orb241331).

### Sequencing and analysis.

Genomes were extracted as previously described (44). Sequencing libraries were made using NexteraXT library preparation kits (FC-131-1096, Illumina). Samples were run paired-end 150-bp reads on the Illumina HiSeq 2500 platform. Reads were aligned to reference genomes for M90T (GenBank accession no. CM001474.1) and the virulence plasmid, pWR501 (GenBank accession no. NC_002698.1), using Bowtie2 (45) and variants were called using GATK (25).

### Bacteria intracellular growth curves.

Intracellular growth curves of S. flexneri were performed as described by Hale and Formal with some modifications (46). Briefly, 24 h prior to assay, Vero cells were plated in Dulbecco’s modified Eagle medium (DMEM) with 5% fetal bovine serum (FBS) to 90% confluence in a 24-well dish. On the day of the assay, overnight bacterial cultures were diluted 1:100 and grown to mid-late log phase. Cultures were centrifuged (5 min at 5,000 × *g*) and resuspended in DMEM with 5% FBS. Vero cells were washed once with PBS prior to infection. Bacteria were added to each well to achieve a MOI of 10 and the plate was centrifuged at 200 rpm for 5 min. Plates were incubated for 30 min at 37°C and 5% CO_2_. Wells were washed three times with PBS, and DMEM with 5% FBS and 100 μg/mL gentamicin was added to each well; after this, plates were incubated at 37°C and 5% CO_2_ for the duration of the assay. At five time points (0, 1, 3, 5, and 7 h), plates were destructively sampled, supernatant was aspirated, wells were washed 3 times with PBS, Vero cells were lysed with sterile, deionized water and gentle pipetting, and bacterial CFU was enumerated via plating on CR-TS agar plates.

### Bacterial plaque assays.

Plaque assays were performed as described by Oaks et al. with some modifications (47). Briefly, 24 h prior to infection, Vero cells were plated to 90% confluence in a 6-well dish in DMEM with 5% FBS. On the day of the assay, overnight bacterial cultures were diluted 1:100 and grown to mid-late log phase. Cultures were centrifuged (5 min at 8,000 × *g*) and resuspended in DMEM with 5% FBS to achieve a range of MOIs between 100 and 0.1. Vero cells were washed once with PBS. Bacterial suspensions were plated on Vero cells and allowed to attach and invade for 1 h at 37°C and 5% CO_2_ with gentle rocking every 15 min. After the incubation, 2× Minimum Essential Media (MEM) complete (10× MEM, 100× l-glutamine, 100× vitamins, 100× nonessential amino acids [NEAA], 7.5% sodium-bicarbonate added to sterile distilled water to a final concentration of 2× with 200 μg/mL gentamicin) was mixed with an equal volume of molten 1% agarose in water and FBS to 5%, added to each well, and allowed to solidify. Plates were incubated for 48 h at 37°C and 5% CO_2_. An additional top layer of 2% neutral red stain (Millipore Sigma N2889) in PBS with 0.5% agarose was added to each well. After 24 h of incubation, plates were visualized for plaques.

### Data availability.

Data are available on Dryad (https://doi.org/10.5061/dryad.mcvdnck0p). Raw sequencing data are available at the National Center for Biotechnology Information Sequence Read Archive (PRJNA713796).
