# Supplementary material for: Selection for Phage Resistance Reduces Virulence of Shigella flexneri
Source: Appl Environ Microbiol. 2022 Jan 25;88(2):e01514-21. doi: 10.1128/AEM.01514-21 (PMC8788674; doi:10.1128/AEM.01514-21)
Supplement: Supplemental file 1 — Fig. S1 to S3, Table S1. Download AEM.01514-21-s0001.pdf, PDF file, 1.0 MB [file aem.01514-21-s0001.pdf]

Supplemental Information

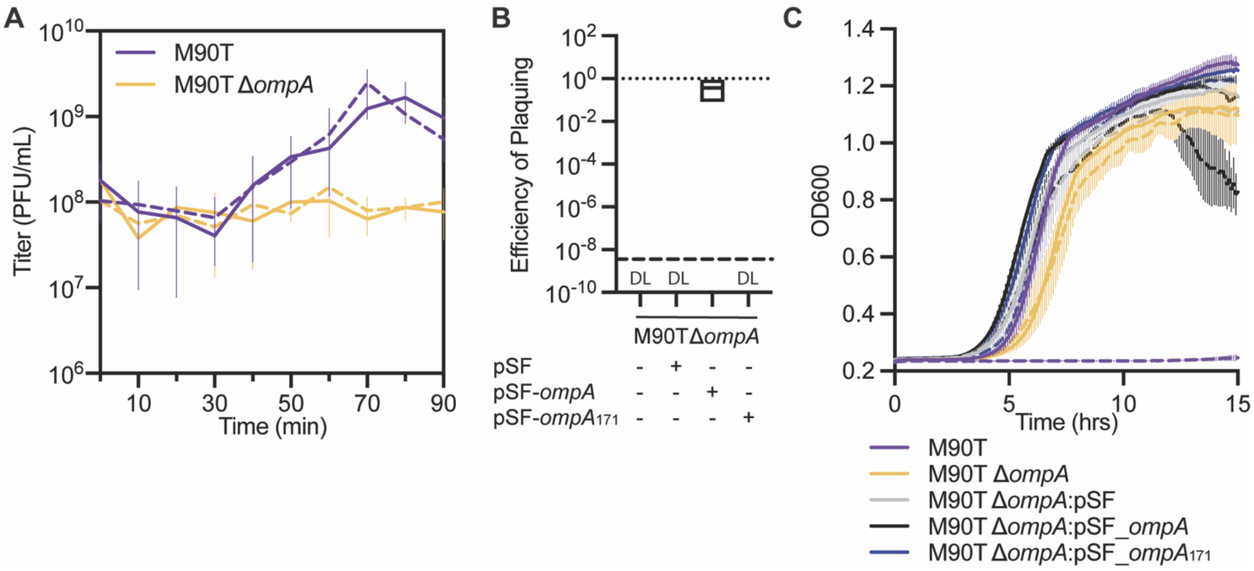

Supplemental Figure S1 A. Phage growth curve of A1-1 on M90T (purple) and M90TΔ*ompA* (yellow). Titers of filtered and chloroformed conditions are indicated with solid and dashed lines respectively. Error bars show standard deviation of three biological replicates. **B.** Efficiency of plaquing (EOP) assay of phage A1-1 on various strains. Dotted line at 1 indicates EOP on M90T. DL indicates that the EOP was below the limit of detection (dashed line). **C.** Bacterial growth curves of M90T (purple), M90TΔ*ompA* (yellow), M90TΔ*ompA*:pSF (grey), M90T Δ*ompA*:pSF_*ompA* (black) and M90TΔ*ompA*:pSF_*ompA*171 (blue). Solid lines indicate bacteria growing in absence of phage; dashed lines indicate phage A1-1 presence (MOI=10).

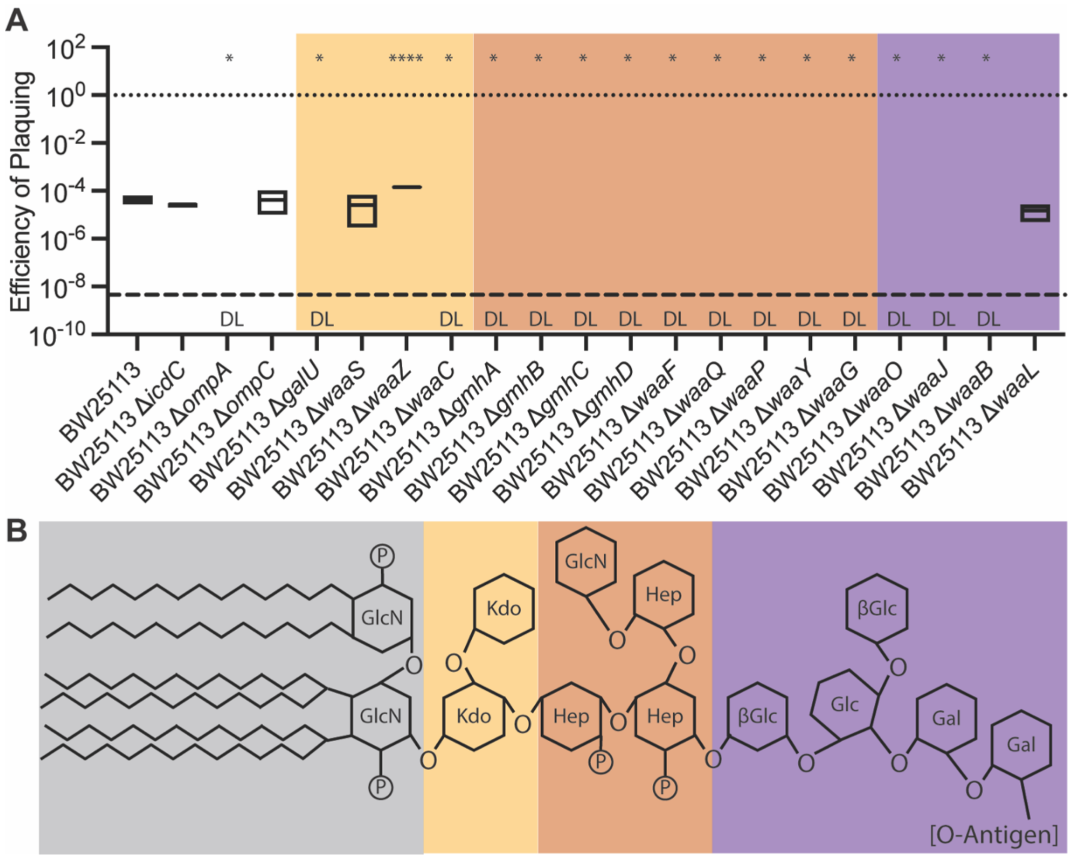

Supplemental Figure S2 A. Efficiency of plaquing (EOP) assay of A1-1 on various strains. Dotted line at 1 indicates EOP on M90T. Yellow shaded knockouts are genes involved in synthesis of KDO sugars. Red shaded knockouts are genes involved in synthesis of the inner core LPS. Purple shaded knockouts are genes involved in synthesis of outer core LPS and O-antigen. Dotted line at 1 indicates EOP on M90T. DL indicates that the EOP was below the limit of detection (dashed line). Significance determined by a one-way ANVOA followed by Dunnet's multiple comparisons test to BW25113. **B.** Diagram of M90T LPS [23] with lipidA (grey), KDO region (yellow), inner core LPS (red) and outer core LPS (purple). Abbreviations as follows phosphoryl group (P), N-acetylglucosamine (GlcN), KDO sugar (Kdo), heptose sugar (Hep), glucose (Glc), and galactose (Gal).

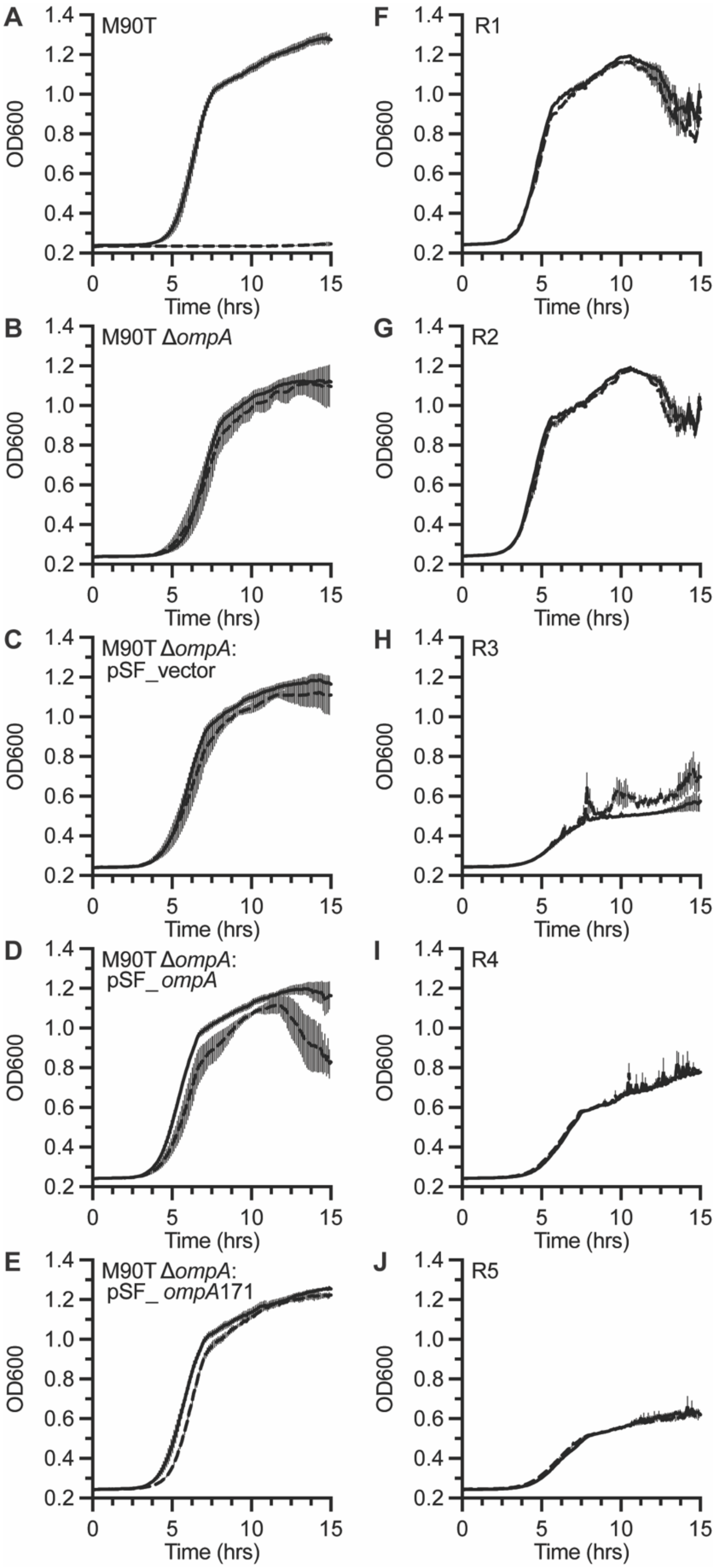

Supplemental Figure S3 Bacterial growth curves of **A.** M90T, **B.** M90TΔ*ompA*, **C.** M90T Δ*ompA*:pSF, **D.** M90T Δ*ompA*:pSF_*ompA*, **E.** M90T Δ*ompA*:pSF_*ompA*171, **F.** R1, **G.** R2, **H.** R3, **I.** R4 and **J.** R5.

Supplemental Table S1 Phage resistance profile of M90T, M90TΔ*ompA*, R1, R2, R3, R4 and R5 to phages 60B and T7. S, phage sensitivity; R, phage resistance.

	Phage 60B	Phage T7
M90T	S	R
M90TΔ*ompA*	S	R
R1	S	R
R2	S	R
R3	R	S
R4	R	S
R5	R	S
